# miR-212-5p attenuates ferroptotic neuronal death after traumatic brain injury by targeting Ptgs2

**DOI:** 10.1186/s13041-019-0501-0

**Published:** 2019-09-18

**Authors:** Xiao Xiao, Youjing Jiang, Weibo Liang, Yanyun Wang, Shuqiang Cao, He Yan, Linbo Gao, Lin Zhang

**Affiliations:** 1 Department of Forensic Genetics, West China School of Basic Medical Sciences & Forensic Medicine, Sichuan University, Chengdu, 610041 Sichuan People’s Republic of China; 2 Laboratory of Molecular Translational Medicine, Center for Translational Medicine, Key Laboratory of Birth Defects and Related Diseases of Women and Children (Sichuan University), Ministry of Education, West China Second University Hospital, Sichuan University, Chengdu, Sichuan People’s Republic of China; 3 Department of Forensic Pathology, West China School of Basic Medical Sciences & Forensic Medicine, Sichuan University, Chengdu, Sichuan People’s Republic of China

**Keywords:** Traumatic brain injury (TBI), Ferroptosis, miR-212-5p, Prostaglandin endoperoxide synthase-2 (Ptgs2)

## Abstract

Ferroptosis, a newly discovered form of iron-dependent regulated cell death, has been implicated in traumatic brain injury (TBI). MiR-212-5p has previously been reported to be downregulated in extracellular vesicles following TBI. To investigate whether miR-212-5p is involved in the ferroptotic neuronal death in TBI mice, we first examined the accumulation of malondialdehyde (MDA) and ferrous ion, and the expression of ferroptosis-related molecules at 6 h, 12 h, 24 h, 48 h and 72 h following controlled cortical impact (CCI) in mice. There was a significant upregulation in the expression of *Gpx4* and *Acsl4* at 6 h, *Slc7a11* from 12 h to 72 h, and *Nox2* and *Sat1* from 6 h to 72 h post injury. Similarly, an upregulation in the expression of Gpx4 at 6 h, Nox2 from 6 h to 72 h, xCT from 12 h to 72 h, and Sat1 at 72 h after CCI was observed at the protein level. Interestingly, MDA and ferrous ion were increased whereas miR-212-5p was decreased in the CCI group compared to the sham group. Furthermore, we found that overexpression of miR-212-5p attenuated ferroptosis while downregulation of miR-212-5p promoted ferroptotic cell death partially by targeting prostaglandin-endoperoxide synthase-2 (Ptgs2) in HT-22 and Neuro-2a cell lines. In addition, administration of miR-212-5p in CCI mice significantly improved learning and spatial memory. Collectively, these findings indicate that miR-212-5p may protect against ferroptotic neuronal death in CCI mice partially by targeting Ptgs2.

## Introduction

Traumatic brain injury (TBI) is defined as an alteration in brain morphology or a disruption in brain function resulting from an external force [1]. As a major public health problem of substantial proportion, TBI and its complications have become a huge burden to individuals, families and society in general [2–5]. It is reported that there are more than 50 million new TBI cases occurring annually, and TBI survivors have a pooled standardized mortality ratio of 2.18 (95% CI 1.88–2.52) according to the global burden of disease (GBD) studies [6]. However, the knowledge development has not caught up with the rising worldwide burden of TBI, most attempts at new medical interventions have failed to show superior efficacy compared with conventional therapies [7]. Thus, both clinical and experimental studies are needed to better understand the mechanism of TBI.

Ferroptosis is a recently recognized type of non-apoptotic cell death which occurs as a consequence of lethal lipid peroxidation [8]. Amino acid, lipid, and iron metabolism are intimately involved in determining cellular sensitivity to the initiation and execution of ferroptosis [8]. Diverse stresses destabilize these metabolic processes, and induce excessive intracellular accumulation of ROS that culminates in the collapse and rupture of the membrane structure of cellular organelles, such as mitochondria, endoplasmic reticulum [9], and lysosome [10]. Recent studies have identified a connection between ferroptosis and the pathophysiology of acute organ damage, like acute lung injury (ALI), acute kidney injury (AKI), and TBI [11, 12]. More importantly, it has been implicated in the pathological process associated with neurodegenerative diseases [13] for which TBI might represent a potential risk factor [14–16]. Given that ferroptosis might be involved in the development of TBI, new approaches, aiming to block ferroptosis-related brain cell death, may help minimize the risk of TBI and maximize chances of recovery.

MicroRNAs (miRNAs) are single stranded RNAs (20–24 nucleotides) that negatively regulate gene expression by binding to the 3′-untranslated region (UTR) of specific target mRNAs, which results in inhibition of mRNA translation or direct degradation of the mRNA [17]. MiRNAs have been shown to be induced by TBI and therefore may offer therapeutic potential [18]. Previous studies have demonstrated that miR-212 is highly expressed in the brain [19] where it may play a role in synaptic plasticity [20], memory formation [21, 22], and blood brain barrier integrity [23]. Meanwhile, this conserved miRNA was shown to be downregulated in Alzheimer’s disease (AD), and the tau protein level was increased in the brain of miR-212 knockout mice [24, 25]. Importantly, miR-212-5p has been reported to be down-regulated in the extracellular vesicles following controlled cortical injury (CCI) [26].

Here, we first set out to demonstrate the occurrence of ferroptosis in TBI through assessing related molecules and investigating iron and malondialdehyde accumulation after TBI. Subsequently, we discovered that miR-212-5p was significantly downregulated after TBI and functionally suppressed ferroptosis by directly targeting Ptgs2. Moreover, we tested the efficacy of miR-212-5p agomir in alleviating the neurobehavioral deficits following TBI in mice. These findings highlight the significance of ferroptosis in TBI and introduce miR-212-5p as a critical regulator of ferroptosis.

## Materials and methods

### Animals and ethics statement

Adult male C57BL/6 J mice, aged 10–12 weeks and weighing 20 to 24 g, were kept in individually ventilated cages receiving food and water ad libitum for at least 7 days prior to operation under standard conditions (12 h light/dark cycle, 22–25 °C, 50% relative humidity). Food was withheld overnight prior to operation. All procedures were approved by the Animal Care and Experimental Committee of Sichuan University.

### CCI model

Mice were subjected to a CCI with craniotomy as previously described [27–29]. Briefly, following anesthesia with isoflurane (4% for induction and 1–2% for maintenance), mice were mounted on a stereotaxic frame. A midsagittal incision was performed in the scalp under sterile conditions and a 4.5 mm diameter circular craniotomy was made over the left parietotemporal cortex with a burr drill. Then, the skullcap was gently removed and a 3.0 mm diameter round and flat tip was carefully placed vertically to the dural surface. The electromagnetic controlled cortical impact device was set to 5.0 m/s for strike velocity, 2.0 mm for strike depth and 100 ms for dwell time. A sterile plastic film covered the bone window and intermittent sutures of the skin, and disinfection with iodophor were performed. The entire procedure required 15–30 min per mouse. Mice with dural lacerations were removed. After a certain survival period, the mice were anesthetized and perfused transcardially with 10 ml of isotonic saline. The ipsilateral cortex around the injury site was rapidly dissected and stored at − 80 °C. In total, 180 mice were randomly divided into two groups: the CCI group; and the sham group that underwent craniotomy without injury. Among them, 150 samples were each cut into two identical pieces (total = 300 pieces) and used for qRT-PCR (*n* = 90 pieces), Western blotting (*n* = 50 pieces), iron (*n* = 60 pieces), and MDA assay (*n* = 60 pieces); the remaining tissues were stored and used for other experiments, while 30 mice were assigned to perform the Morris water maze (MWM) test. Four dead or model failure mice were excluded (CCI: 2/90; sham: 2/90).

### Iron assay

Ferrous ion (Fe^2+^) level in tissues were estimated by the iron assay kit (#ab83366, Abcam, Cambridge, Mass, USA) according to the manufacturer’s instructions. In brief, after washing in ice-cold PBS, tissue samples were weighed and homogenized in 4–10 X volumes of iron assay buffer. Then, iron reducer was added to each supernatant. The mixture was incubated for 30 min, and the output was promptly assessed on a colorimetric microplate reader (OD = 593 nm). The Fe^2+^ level was normalized to the weight of the tissue samples.

### MDA assay

The content of MDA in tissue samples was determined using a Lipid Peroxidation (MDA) Assay Kit (#S0131, Beyotime, Haimen, Jiangsu, China) according to the manufacturer’s instructions. In brief, the tissue samples were weighed and homogenized in ice-cold PBS containing 1% phenylmethanesulfonyl fluoride (PMSF) on ice. Tissue lysates were then centrifuged at 12,000 g for 15 min at 4 °C to collect the supernatant. The MDA in the sample reacted with thiobarbituric acid (TBA) to generate an MDA-TBA adduct that was colorimetrically quantified (OD = 532 nm). The MDA level was normalized to the weight of tissue samples.

### Chemicals and antibodies

Rsl3 (#S8155) and ferrostatin-1 (Fer-1, #S7243) were purchased from Selleck Chemicals (Houston, TX, USA), while Necrosulfonamide (ab143839) and Z-VAD-FMK (ab120382) were purchased from Abcam (Cambridge, Mass, USA). The antibodies used in this study were as follows: Gpx4 (ab125066, Abcam, Cambridge, Mass, USA), Acsl4 (ab137525, Abcam), xCT (ab175186, Abcam), Nox2 (Cybb, ab80508, Abcam), Sat1 (PA1–16992, Thermo, Rochester, NY, *USA*), Actb (Lot#Nb15, Absin, Shanghai, China). HRP-conjugated secondary antibody was obtained from ZSGB-BIO (Beijing, China).

### Cell culture

The immortalized mouse hippocampal neuron line (HT22) and the mouse neuroblastoma cell line (Neuro-2a) were obtained from Honsun Biological Technology (Shanghai, China) and were cultured in Dulbecco’s Modified Eagle’s Medium (DMEM, Hyclone, Utah, USA) supplemented with 10% fetal bovine serum (Hyclone, Utah, USA), and 1% penicillin–streptomycin.

### RNA extraction, cDNA synthesis and real-time PCR analysis

Quantitative real-time PCR (qRT-PCR) was performed to assess the expression level of miR-212-5p and the mRNA expression of the ferroptosis-related molecules *Gpx4, Acsl4, Slc7a11, Nox2, Sat1, Ptgs2* at 6 h, 12 h, 24 h, 48 h, and 72 h following CCI. In brief, total RNA was prepared from cortical tissue samples using the Trizol reagent (Invitrogen, Carlsbad, CA, USA). The cDNA synthesis was achieved with RevertAid First Strand cDNA Synthesis Kit (#K1621, Thermo Scientific, USA) after determining the quantity of total RNA with a NanoDrop ND-1000 (NanoDrop, Wilmington, DE). qRT-PCR using the SYBR™ Green PCR Master Mix (#43091055, ThermoFisher, USA) was then conducted on a Life Technologies Prism 7500 instrument (Life Technologies, Foster City, CA, USA). For the microRNA, a similar procedure was performed except that the cDNA synthesis and qRT-PCR was conducted applying miDETECT A Track™ miRNA qRT-PCR Starter Kit (#R11068.5, RiboBio, Guangzhou, Guangdong, China). *β-actin* (Actb) and noncoding small nuclear RNA (U6) were applied as internal controls respectively to normalize the data. The primers are available upon request.

### Western blotting

Cortical tissue samples were isolated and lysed in RIPA reagent (Beyotime, Haimen, Jiangsu, China) containing 1% PMSF (Beyotime, Haimen, Jiangsu, China). BCA assay kit (Beyotime, Shanghai, China) was then used to quantified the total protein extraction. Protein samples were separated on a 10% SDS polyacrylamide gel by electrophoresis, and then electro-transferred onto polyvinylidene difluoride membranes (Bio-Rad Laboratories, Hercules, CA, USA). The membranes were incubated with primary antibodies at 4 °C overnight after blocking with bovine serum albumin at room temperature for 1 h. The membranes were then incubated with secondary antibodies after washing in Tris-buffered saline with Tween 20 (TBST). Finally, signals were visualized by an ECL kit (Merck millipore, Massachusetts, USA). NIH Image J software (Bethesda, MD, USA) was applied to quantify the band density, while β-actin was used as the loading control.

### Mimic, inhibitor and transfection

The mmu-miR-212-5p mimic, inhibitor and their negative controls were purchased from RiboBio (#R10034.8, Guangzhou, Guangdong, China). Cells were dissociated using 0.05% trypsin and counted with a Neubauer hemocytometer. Transfections were performed according to the manufacturer’s instructions with Lipofectamine 3000 (L3000015, Invitrogen, Carlsbad, CA, USA). Briefly, the mimic, inhibitor (20 μM) and transfection reagent were diluted in Opti-MEM medium. After mixing and incubating at room temperature for 15 min, the solution was transfected into HT22 and Neuro-2a cells.

### Cell death assay

Lactate dehydrogenase (LDH) activity was measured to determine the cell death using the Cytotoxicity Detection Kit (#G1780, Promega, Madison, Wi, USA). Prior to treatment, cells were planted into 96 well culture plate and Rsl3 was applied at a final concentration of 3 μM in the culture medium for 24 h. To elucidate the specific induction of ferroptosis, different cell death inhibitors combined with RSL3 were used, including ferrostatin-1, necrosulfonamide, and zVAD-fmk. Incubation conditions: ferrostatin-1, 1 μM; zVAD-fmk, 10 μM; and necrosulfonamide, 0.5 μM.

### Plasmid construction and luciferase reporter assay

The 3′-UTR of *Ptgs2* mRNA with potential target sites of miR-212-5p was amplified through PCR from gDNA. The amplicon was inserted into a pGL3 basic vector with *Xba* I and *Xho* I restriction enzyme sites (Promega, Madison, WI, USA) (forward primer, 5′-ACTCGAGGCCAGTGAGAAGGGAAATGA-3′ and reverse primer, 5′-CCTCTAGATGAACTTGGACCCCTTTGTT-3′). Subsequently, plasmid DNA (pGL3-Ptgs2-wt) was isolated from recombinant colonies and verified by sequencing. To generate the 3′-UTR mutants of Ptgs2 (pGL3-Ptgs2-mt), the binding site (CCAAGG) was altered to (TGCCAC) using the site-directed mutagenesis kit (NEB E0554, Grand Island, Nebraska, USA), and sequenced to guarantee its authenticity. HT22 and Neuro-2a cells were co-transfected with empty, wild-type (pGL3-Ptgs2-wt) or mutant-type (pGL3-Ptgs2-mt) reporter vectors and miR-212-5p mimic by Lipofectamine 3000 reagent. Obtained 48 h after transfection, cells were examined with the dual-luciferase reporter assay (Cat. #E1960, Promega, Madison, Wi, USA). Three independent experiments with at least two replicates were applied and Renilla luciferase was used to normalize the transfection efficiency.

### Intracerebroventricular injection (ICV)

The agomir and negative control (NC) were purchased from RiboBio (Guangzhou, Guangdong, China). Anesthetized mice were placed in the stereotaxic frame. With a 5-μl-gauge hamilton-syringe, 10 μl of 5 nM agomir or NC were stereotactically delivered into the right lateral ventricle at 1 μl/min for 10 min. The syringe was positioned at the following coordinates: AP − 0.3 mm, ML + 0.9 mm, and DV − 2.8 mm. On the 4th and 2nd day prior to TBI, the agomir or NC was consecutively injected in order to ensure adequate concentration and efficacy.

### Morris water maze

To evaluate memory and spatial learning of mice following CCI, the MWM test was performed as previously described [30, 31]. A plastic tank (85 cm in diameter and 60 cm in height) located in a room with four visual cues was filled with water (23 °C ± 1 °C) to a height of 30 cm. The MWM test began on the 7th day following CCI. Briefly, mice were individually placed in the apparatus in one of the four different quadrants and allowed up to 60s to reach the submersed platform in the place navigation test. Mice were guided to the submersed platform if they failed to find it, and all of them were allowed to remain on the platform for an additional 10s. The behavioral data were automatically recorded and studied using a video tracking system (#WMT-100S, Techman soft, Chengdu, Sichuan, China). On the 15th day, the spatial probe test was conducted without the hidden platform. During the test, the frequency of crossing the platform location was recorded and analyzed by the same video computer system.

### Statistical analyses

Data are presented as means ± standard error of the mean (SEM). A Student’s *t*-test was used to compare means of the two groups. One-way analysis of variance (ANOVA) was used for comparisons among multiple groups and Tukey’s post hoc test was applied to identify specific differences between groups, while the data of escape latency was analyzed using repeated-measures two-way analysis of variance (ANOVA). All data analysis was done using GraphPad Prism 7.0 (GraphPad Software, Inc., Cary, NC). A value of *p* < 0.05 is considered to be statistically significant.

## Result

### CCI is followed by changes in lipid formation, ferrous ion levels and expression of ferroptosis-related molecules

**Fig. 1:**
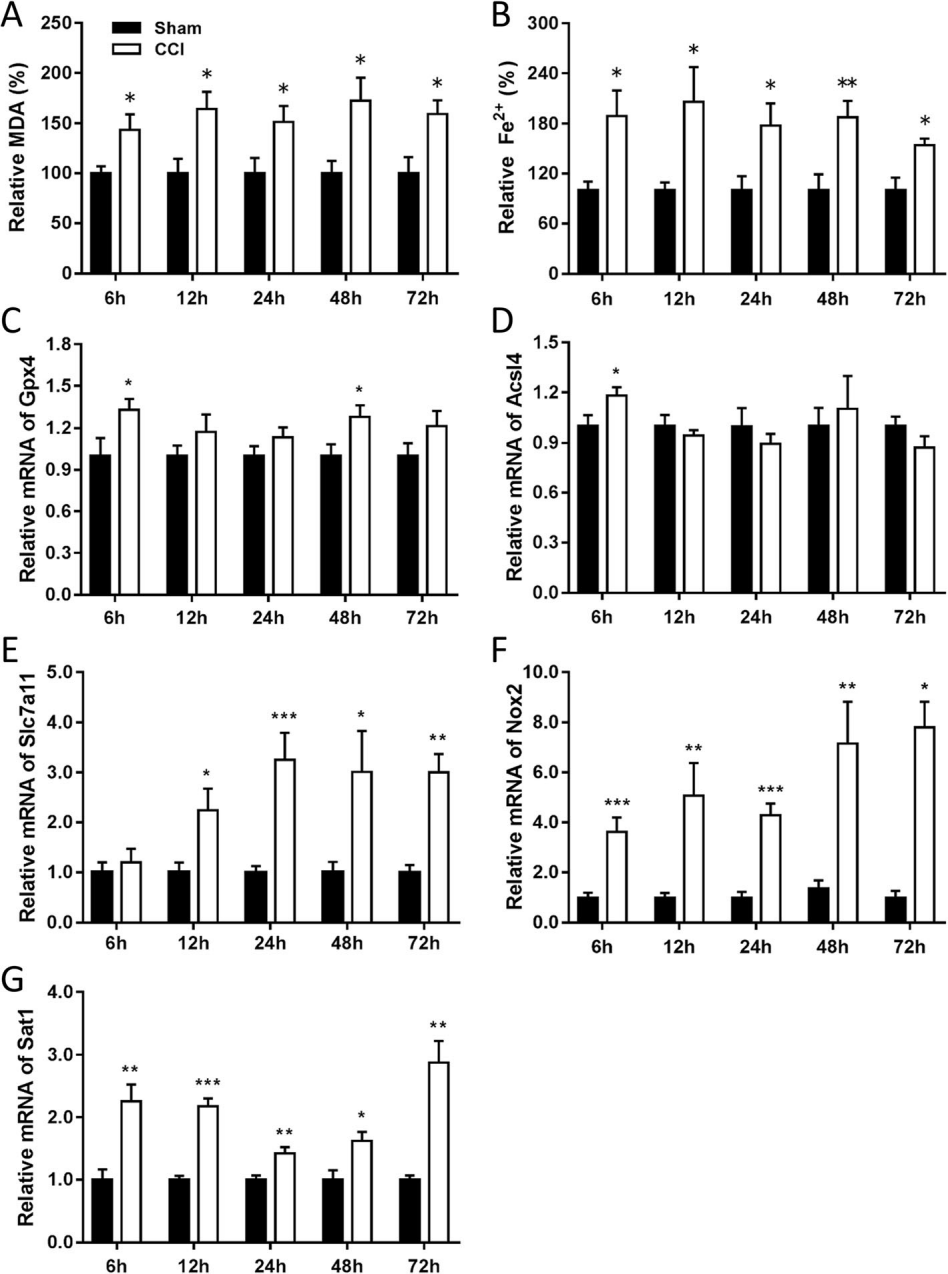
The concentration of MDA and Fe^2+^ and the mRNA levels of ferroptosis-related molecules following CCI. MDA (**a**) and Fe^2+^ (**b**) levels in the ipsilateral cortex at 6 h, 12 h, 24 h, 48 h, and 72 h after CCI (*n* = 6 for each group). Quantitative RT-PCR showing the relative expression levels of *Gpx4* (**c**), *Acsl4* (**d**), *Slc7a11* (**e**), *Nox2* (**f**), *Sat1* (**g**) in the cortex at the indicated times after injury (*n* = 9 for each group). Data are expressed as mean ± SEM. Data were analyzed using the student’s *t*-test. *p < 0.05, **p < 0.01, ***p < 0.001. MDA, malondialdehyde; CCI, controlled cortical impact; Fe^2+^, ferrous ion

**Fig. 2:**
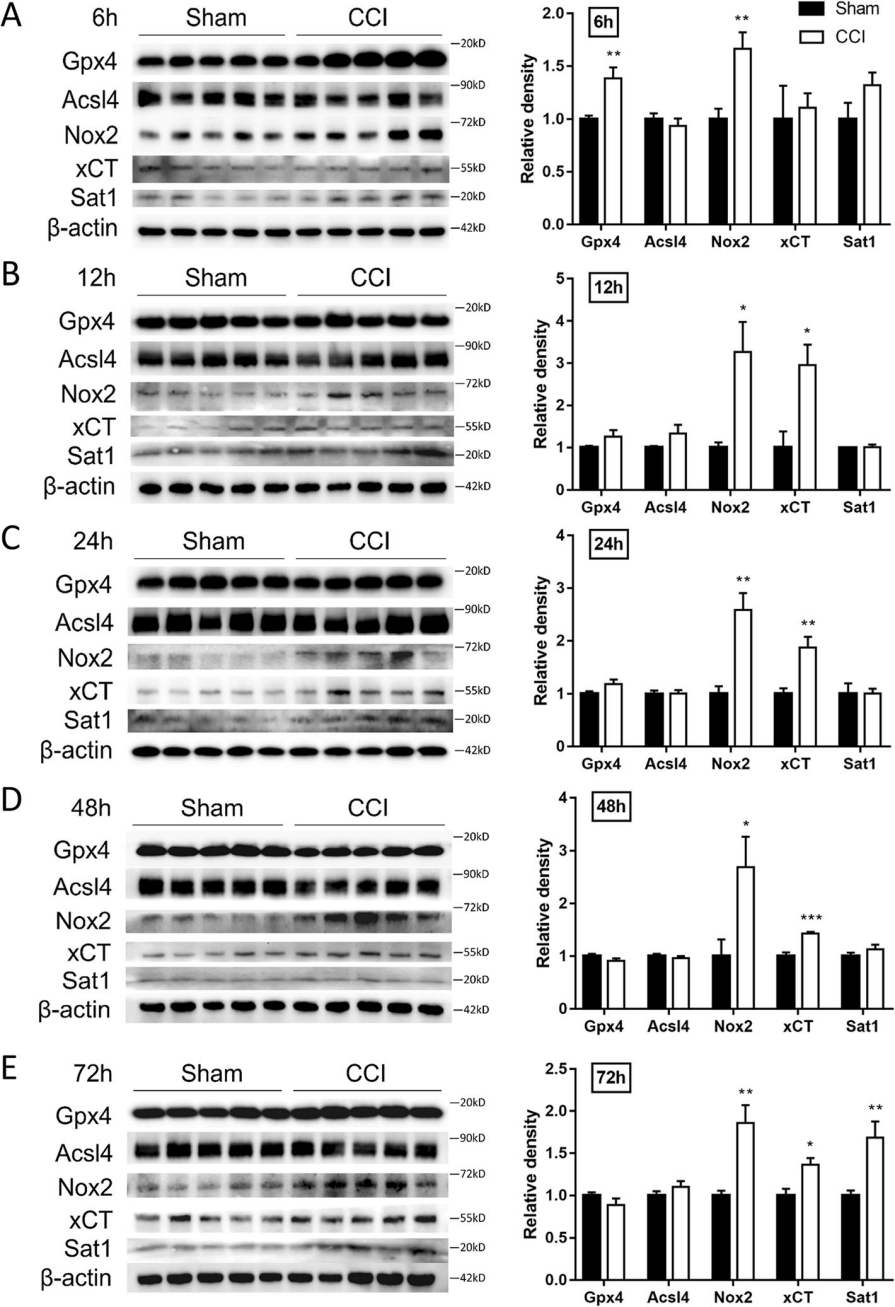
The protein levels of ferroptosis-related molecules at the indicated times after CCI. Cortical expression of Gpx4, Acsl4, Nox2, xCT and Sat1 were measured in CCI and sham groups at 6 h (**a**), 12 h (**b**), 24 h (**c**), 48 h (**d**), 72 h (**e**). Data are expressed as mean ± SEM and analyzed with the student’s *t*-test, *n* = 5 per group, *p < 0.05, **p < 0.01, ***p < 0.001. CCI, controlled cortical impact

MDA is an end-product of polyunsaturated fatty acid peroxidation, while the level of Fe^2+^ reflects the iron overload which has the potential to generate highly reactive hydroxyl radicals through the fenton reaction [32]. We firstly demonstrated CCI results in the accumulation of these two markers. As shown in Fig. 1a and b, both the levels of ferrous ion and MDA were significantly elevated at 6 h, 12 h, 24 h, 48 h, and 72 h post injury. The genes essential for ferroptosis regulation were selected to evaluate mRNA and protein levels using qRT-PCR and Western-blotting, including Gpx4 (protects cells against membrane lipid peroxidation), Acsl4 (related to the generation of lipid peroxidation products), xCT (a sodium-independent cystine-glutamate antiporter) and Sat1 (involved in p53-mediated ferroptosis). The mRNA levels of *Gpx4* (Fig. 1c) and *Acsl4* (Fig. 1d) were upregulated at 6 h post injury, while the mRNA levels of *Slc7a11* (Fig. 1e) were upregulated from 12 h to 72 h post injury. The mRNA levels of *Nox2* (Fig. 1f) and *Sat1* (Fig. 1g) were constantly upregulated from 6 h to 72 h. The protein levels were also observed to be significantly upregulated, with Gpx4 at 6 h, Nox2 from 6 h to 72 h, xCT from 12 h to 72 h, and Sat1 at 72 h following CCI (Fig. 2).

### MiR-212-5p is downregulated after TBI and regulates Rsl3-induced ferroptosis in HT22 and neuro-2a cells

**Fig. 3:**
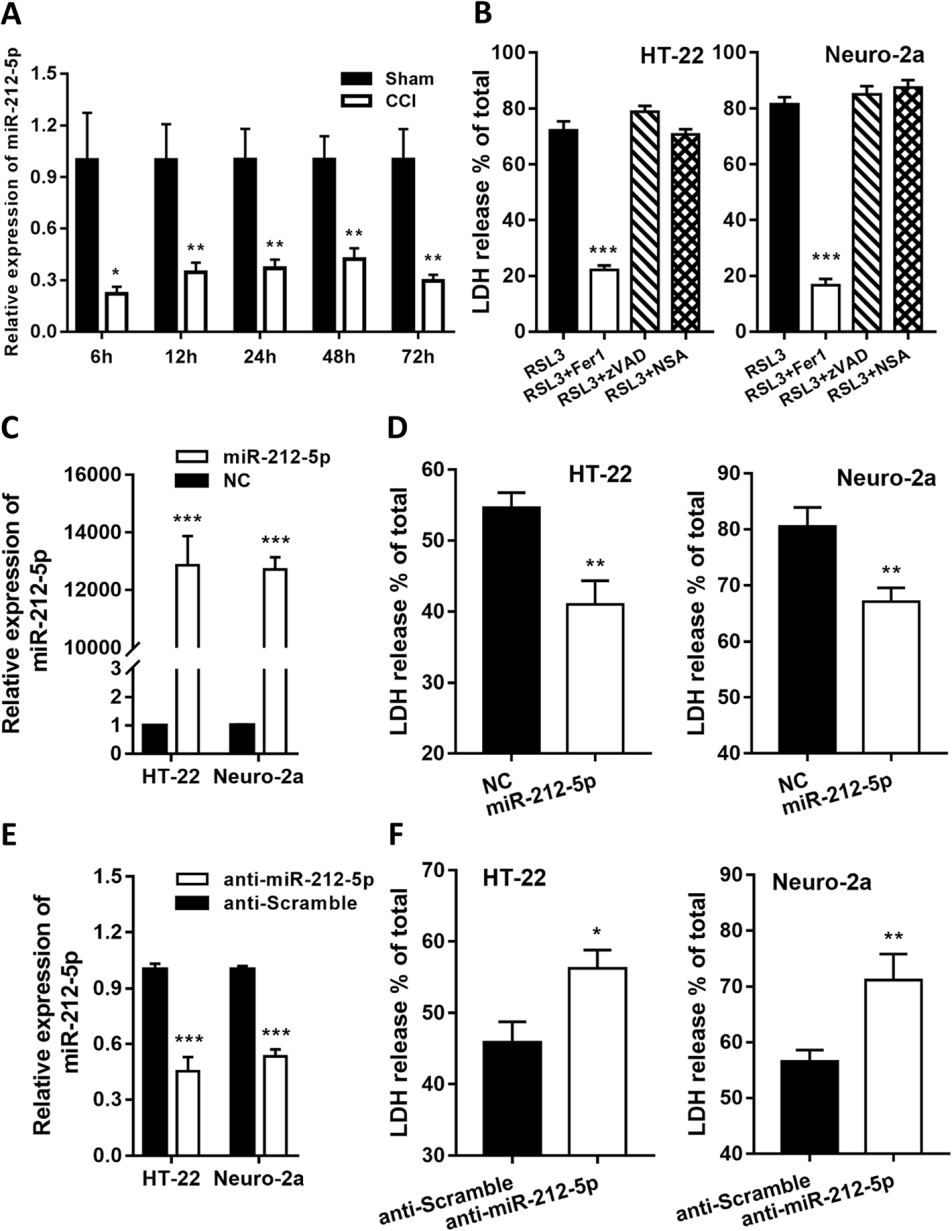
miR-212-5p was constantly downregulated after CCI and negatively regulated ferroptosis in neuronal cell lines. **a** Quantitative RT-PCR showing the relative expression levels of miR-212-5p in the cortex at 6 h, 12 h, 1d, 2d, 3d after injury (*n* = 9 for each group). **b** The HT-22 and Neuro-2a cells were treated with Rsl3 (3 μM) with or without a cell death inhibitor (ferrostatin-1, 1 μM; zVAD-fmk, 10 μM; necrosulfonamide, 0.5 μM) for 24 h. Data were analyzed using analysis of variance (ANOVA) with Tukey’s post hoc test. **c** Overexpression of miR-212-5p suppressed Rsl3-induced cell death in HT-22 and Neuro-2a cells. **d** Inhibition of endogenous miR-212-5p enhanced Rsl3-induced cell death in HT-22 and Neuro-2a cells. Indicated cells were treated with Rsl3 (3 μM) for 24 h. The transfection efficacy of overexpression (**e**) or inhibition (**f**) was confirmed by qRT-PCR analysis. Cell death was assayed using the Cytotoxicity Detection kit. Data shown represent mean ± SEM from three independent experiments. *p < 0.05, **p < 0.01, ***p < 0.001. CCI, controlled cortical impact

Previous studies have reported that CCI significantly decreased the level of miR-212-5p in the extracellular vesicles of brain tissue. We confirmed that the expression of miR-212-5p was constantly downregulated at 6 h, 12 h, 24 h, 48 h, 72 h after TBI (Fig. 3a), and the reduction was significant, for its expression was shrunk, on an average, by 66% relative to the sham group at most indicated time points.

To elucidate whether miR-212-5p can influence ferroptotic neuronal death, we first assessed the ferroptosis-inducing activity of Rsl3 in HT-22 and Neuro-2a cell lines. Rsl3 successfully induced cell death in both cell lines and this process was reversed by using the inhibitor of ferroptosis, Fer-1 (Fig. 3b). Contrarily, no reverse effects were observed on Rsl3-induced cell death with treatment of the apoptosis inhibitor zVAD-fmk or necroptosis inhibitor necrosulfonamide (Fig. 3b). Taken together, these findings imply that ferroptosis can be sufficiently induced by Rsl3 in both HT-22 and Neuro-2a cell lines.

Thereafter, we overexpressed miR-212-5p by transfecting HT-22 and Neuro-2a cells with a miR-212-5p mimic (Fig. 3c) and suppressed it by applying a miR-212-5p inhibitor (Fig. 3d). Compared to the cells transfected with the mimic negative control, overexpression of miR-212-5p decreased the cell death rate (Fig. 3e) while suppression of miR-212-5p expression increased the cell death rate (Fig. 3f). These results indicate that miR-212-5p plays a critical role in regulating ferroptosis in HT-22 and Neuro-2a cells.

### MiR-212-5p negatively regulates Ptgs2

**Fig. 4:**
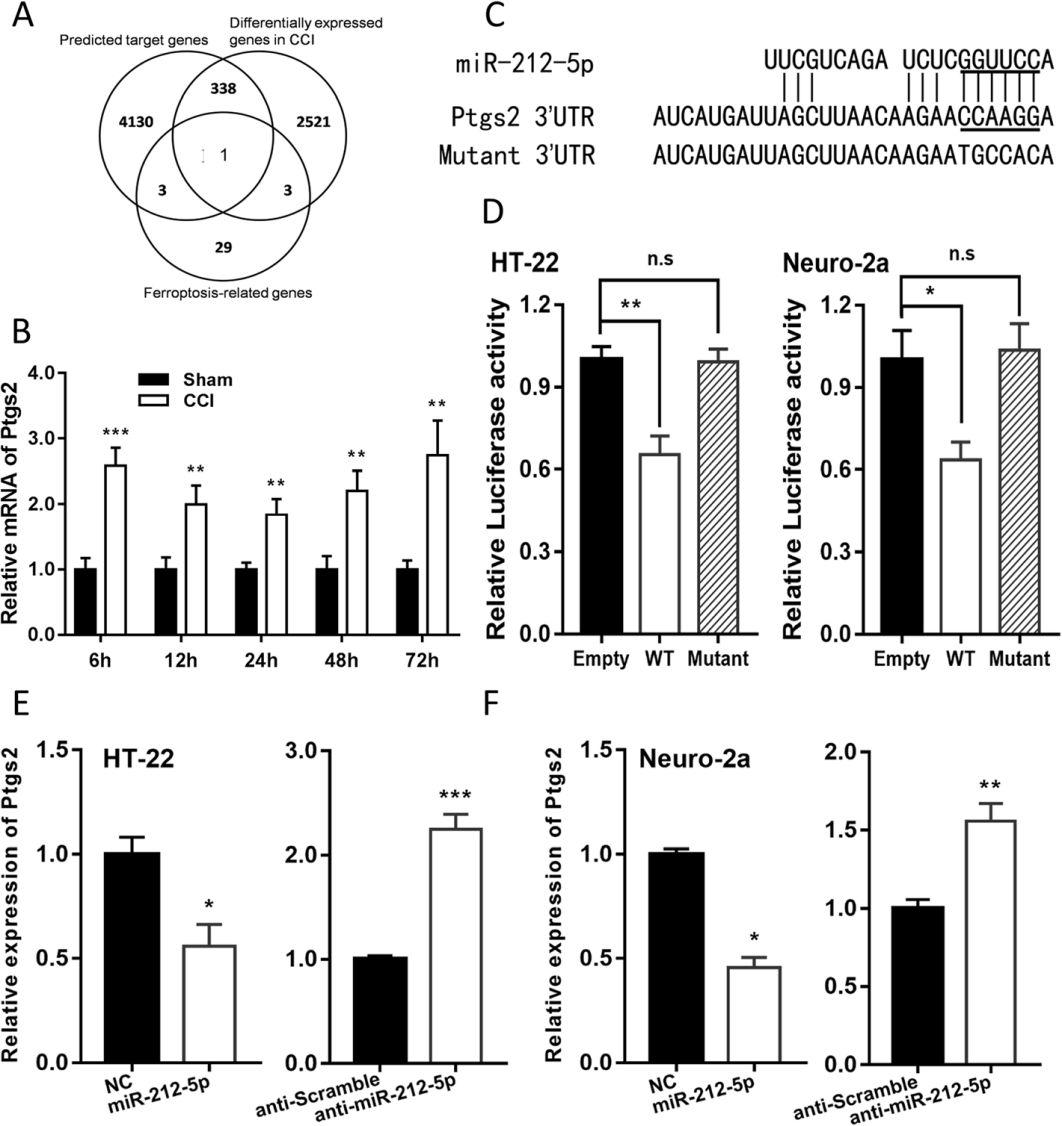
miR-212-5p directly targets Ptgs2 in HT22 and Neuro-2a cell lines. Schematic diagram of strategy to discover the target gene of miR-212-5p is shown in (**a**). **b** The expression of Ptgs2 was significantly increased at all indicated time points after CCI. **c** Sequence alignment of miR-212-5p and the 3′-UTR of Ptgs2. The seed sequence of miR-212-5p and the binding sites in 3′-UTR are marked with underscores. **d** miR-212-5p overexpression inhibited the expression of 3′-UTR-luciferase reporter of Ptgs2 in HT-22 and Neuro-2a cells, but the mutant construct was immune to miR-212-5p. The 3′-UTR mutants containing mismatched nucleotides are shown at the bottom. In HT-22 (**e**) or Neuro-2a (**f**) cells, miR-212-5p overexpression inhibited the expression of Ptgs2, while inhibition of miR-212-5p caused the downregulation of Ptgs2. Data are presented as mean ± SEM from three independent experiments. *p < 0.05, **p < 0.01, ***p < 0.001

To identify a specific target of miR-212-5p that may be implicated in both the ferroptosis pathway and the pathological process of TBI pathway, we investigated mRNAs that are differentially expressed following TBI from a publicly available transcriptional profile, GSE79441 from http://www.ncbi.nlm.nih.gov/geo, genes with potential binding sites of miR-212-5p using bioinformatics tool *miRwalk* [33], and molecules involved in ferroptosis [8, 9]. One single gene, Ptgs2, emerged in all three categories (Fig. 4a) and we experimentally observed cortical Ptgs2 to be upregulated in CCI mice than the sham groups at all indicated time points (Fig. 4b).

To confirm the bioinformatics prediction, dual-luciferase activity assay was applied in HT-22 and Neuro-2a cells. Firstly, the 3′-UTR sequence of Ptgs2 was cloned into the pGL3 basic vector and some mismatch mutations were introduced into the seed sequences in order to create mutant reporter vectors (Fig. 4c). The luciferase activity of pGL3-Ptgs2-wt vector was significantly decreased by cotransfection of HT-22 and Neuro-2a cells with miR-212-5p mimic, while the administration of pGL3-Ptgs2-mt vector reversed the inhibitory effect of miR-212-5p (Fig. 4d). These findings indicate that the 3′-UTR of Ptgs2 is a direct target of miR-212-5p.

Furthermore, the mRNA level of Ptgs2 was analyzed by qRT-PCR in HT-22 and Neuro-2a cells transfected with miR-212-5p mimic and inhibitor. We found that cells transfected with the mimic led to a significant downregulation in mRNA level of Ptgs2, while treatment with miR-212-5p inhibitor conversely caused an upregulated mRNA expression of Ptgs2 (Fig. 4e and f). According to the above-mentioned results, miR-212-5p regulates mRNA levels of Ptgs2 in both HT-22 and Neuro-2a cells, and suppresses the ferroptotic neuronal death partly by targeting Ptgs2.

### Overexpression of miR-212-5p improves performance in Morris water maze test following CCI in mice

**Fig. 5:**
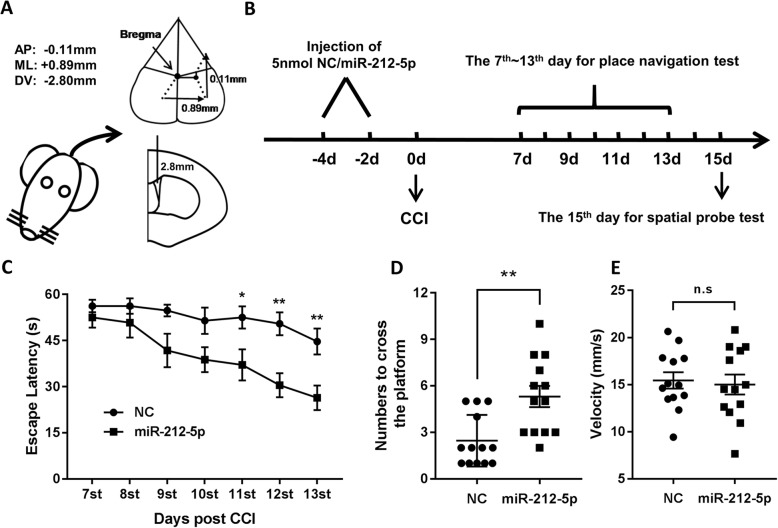
miR-212-5p improved water maze performance after CCI in mice. **a** Intracerebroventricular (i.c.v.) injection site brain schematic diagram. **b** time course of the experiments. **c** in MWM test, mean escape latency for each group was plotted during the 7th to 13th day. Data of escape latency are performed using two-way analysis of variance (ANOVA) for repeated measures. **d** The frequency of crossing the platform was recorded on the 15th day. The speed of each mouse in the spatial probe test was recorded and shown in (**e**). These Data are presented as mean ± SEM (*n* = 13 for each group) and analyzed with the student’s *t*-test. *p < 0.05, **p < 0.01. CCI, controlled cortical impact; MWM, Morris Water Maze

To determine the protective effect of miR-212-5p on the neurological deficits in CCI mice, the MWM test was conducted to assess spatial learning and memory ability. For this purpose, we stereotactically injected miR-212-5p agomir into the right ventricle of mice prior to TBI induction (Fig. 5a and b). MiR-212-5p agomir pretreatment remarkably shortened the escape latencies of MWM on days 11–13 in CCI mice (Fig. 5c). For the spatial probe test, frequency of passing through the platform quadrant of the pretreatment group was significantly higher than that of the CCI group (Fig. 5d) with relatively equivalent swimming speeds (Fig. 5f). Our results imply that intracerebroventricular administration of miR-212-5p may be advantageous to memory and learning improvement in CCI mice.

## Discussion

As a distinct form of regulated cell death, ferroptosis has several connections to disease pathogenesis, including cancer, stroke and stress disorders [34]. Although related results are still preliminary, emerging evidence suggests that ferroptosis might also be involved in trauma-related diseases, including acute lung injury, acute kidney failure and traumatic brain injury [8, 12]. Ferroptotic cell death lies at the intersection of amino acid, lipid and iron metabolism [8]. In our study, we observed accumulation of MDA and Fe^2+^ and alternation of the above-mentioned metabolism-related molecules after CCI. Our findings together with previous reports indicate that ferroptosis is involved in the pathogenesis of CCI [35]. Many of our results are consistent with previous studies [11, 35, 36], with some exceptions. Gpx4 (converts toxic lipid hydro-peroxides to non-toxic lipid alcohols) was reported to decrease after TBI [10], but in our study, its expression was barely changed compared to the sham group at all indicated time points except for the 6 h group in which an increased level of Gpx4 was observed. The reason underlying this difference might associate with different control groups that were used, as we compared the CCI groups with the sham operated ones instead of naive groups in which the mice were not subjected to craniotomy. Different ages or species of animals used might also contribute to the differences. As the GPx activity was shown to decrease after TBI [10], it was possibly a compensatory up-regulation of Gpx4. Another study claimed that MDA levels were highly elevated around the lesion during 72 h after TBI [35], but the elevation in our study was not as significant. This difference could partly be attributed to the different settings of sham groups, as we utilized time points-matched sham groups instead of a single sham group. From our results, it appears that the craniotomy itself may serve a role in the pathological process after CCI, thus it may not be appropriate to compare biochemical indicators of the CCI group at different time points without a corresponding sham group. Interestingly, it was observed that a few molecules, such as Gpx4 and Acsl4, relevant to ferroptotic cell death, were differentially expressed only at the early time point after TBI, indicating that ferroptosis might initiate in the early stage of injury, and with the most active phase. As the master regulator of ferroptosis has not been identified [37–39], further research is needed to explore the specific marker and the comprehensive pathological contexts of ferroptosis during TBI.

Previous studies implied that miR-212 is widely involved in the neuronal system [19]. It was related to the regeneration of endothelial progenitor cells [40], integrity of the blood brain barrier [23], dendritic morphology [41] and oligodendrocyte differentiation [42]. It has also been implicated in addiction-related and mental diseases [43–45]. More importantly, miR-132/212 and its corresponding target mRNAs were reported to be involved in the pathogenesis of AD, which was shown to be related to ferroptosis [24, 46, 47].

Our study, to our knowledge, is the first to report the role of miR-212-5p in the ferroptotic neuronal death after TBI, and we identified Ptgs2 as a target gene. Ptgs2, also known as cyclooxygenase-2 (Cox-2), is the key enzyme in prostaglandin biosynthesis, and acts both as a peroxidase and as a dioxygenase [48, 49]. We showed that Ptgs2 is involved in the process of ferroptosis because it was significantly up-regulated after treatment with Rsl3 and erastin in mice [50–52]. These two small molecules were shown to be specific inducers of ferroptotic cell death and ideal probes to elucidate downstream regulators of ferroptosis [53–55]. As a matter of fact, cyclooxygenases can catalyze lipid oxidation [56, 57], however, to date, only the lipoxygenase family has been demonstrated in ferroptosis [8, 36]. Although, the exact role of Ptgs2 in the ferroptotic cell death cascade remains to be elucidated, inhibiting Ptgs2, as induced by ferroptosis, was an effective way to alleviate cell death. According to our results, the expression of miR-212-5p after TBI was significantly downregulated at all indicated time points, and miR-212-5p suppressed ferroptosis in cell lines partly by targeting Ptgs2. These results indicate that miR-212-5p may represent a potential therapeutic target in TBI patients.

Except for acute injuries, ferroptosis has been shown to play an important role in neurodegenerative diseases [58–60], like AD, while evidence is accumulating that TBI may raise the risk of AD [61, 62]. Along with the evidence that miR-132/212 was consistently downregulated in AD [24], it is reasonable to speculate that miR-212 may act like a bridge between TBI and neurodegenerative diseases. Moreover, there is a consensus that crosstalk widely exists between ferroptosis and other forms of regulated cell death [53, 63–66], and emerging evidence indicates that miR-212 may prevent neuron death by inhibiting other target genes, like *Sirt2* [67] or participating in other pathways, like autophagy [65, 66] or apoptosis [63]. Further studies are urgently required to identify whether miR-212-5p is involved in any other pathologic process of TBI.

Previously, extensive studies in vivo focused on the role of miR-212-5p in tumor pathogenesis [68, 69], lipid metabolism [70, 71] and endocrine signaling axis [72, 73], while very limited data reported in nervous system. Sun et al. reported that miR-212-5p could attenuate damage and loss of dopaminergic neuron in the mouse model of Parkinson’s disease [67]; Smith and the colleagues reported that miR-132/212 knockout mice have impaired memory while treatment with miR-132 rescues the memory deficits by reducing the phosphorylated tau [24]. We proved, in this study, the relationship between miR-212-5p and ferroptosis in vitro. Further studies are warranted to identify the cell type-specific expression patterns of miR-212-5p and its function in vivo following CCI for a better discovery of its therapeutic value.

Collectively, our data provides sufficient evidence to confirm the involvement of ferroptosis in CCI by demonstrating that miR-212-5p negatively regulates ferroptosis partially by targeting Ptgs2 in CCI mice. Thus, these findings implicate miR-212-5p as a potential candidate for protecting the impaired brain by attenuating ferroptosis.

## Data Availability

The data in our study are available from the corresponding author upon reasonable request.

## Abbreviations

AD: Alzheimer’s disease
AKI: Acute kidney injury
ALI: Acute lung injury
CCI: Controlled cortical impact
Cox-2: Cyclooxygenase-2
DMEM: Dulbecco’s Modified Eagle’s Medium
Fe^2+^: Ferrous ion
GBD: Global burden of disease
ICV: Intracerebroventricular injection
LDH: Lactate dehydrogenase
MDA: Malondialdehyde
miRNAs: MicroRNAs
MWM: Morris water maze
NC: Negative control
PMSF: Phenylmethanesulfonyl fluoride
Ptgs2: Prostaglandin-endoperoxide synthase-2
qRT-PCR: quantitative real-time PCR
SEM: Standard error of the mean
TBA: Thiobarbituric acid
TBI: Traumatic brain injury
TBST: Tris-buffered saline with Tween 20
UTR: Untranslated region
